# Dr. Dalton’s Lectures on the Physiology of the Circulation

**Published:** 1860-05

**Authors:** 


### EDITORIAL DEPARTMENT.

— With the present number of the Monthly we commence the publication of a series of lectures upon the *Physiology of the Circulation*, by the distinguished physiologist, Prof. John C. Dalton. Four lectures have already appeared in the late *Buffalo Medical Journal*, the fifth appearing in the present number of the united journals. The former subscribers to the old Buffalo Journal will receive the lectures in regular course; and the subscribers of the Monthly, in order to place them upon an equal footing, will receive the four lectures in one extra number, as a supplement to, and paged consecutively with, the April number, so that it can be bound up with the Thirteenth volume.

The republication of these lectures has been attended by considerable expense, but this has been cheerfully assumed, believing, as we do, that we are making by this publication an important addition to the medical literature of the country, and giving to our readers a series of lectures which will prove in every way acceptable and instructive.

Of the lectures themselves it is not necessary for us to say a single word. The high reputation the author enjoys, his devotedness to physiological pursuits, and the eminent rank he has already attained as a physiologist, is the best guarantee of their great value. The Extra April number, containing the first four lectures, comprising 84 pages, will be sent to *paying* subscribers *only.* Those, therefore, who have already paid, may expect to receive this number; and those who may hereafter oblige us by becoming paying subscribers, will also promptly receive this number upon the receipt of their arrearages

It is seldom we refer to pecuniary matters in the pages of our journal. The opportunity is, however, so good, that we cannot refrain from requesting our delinquent subscribers to pay promptly, both as a compliment to us, and as a duty to us. We are striving to make the Monthly as practical, as instructive, and at the same time, as entertaining a journal, as can be found in the country; and we shall look to all those who have subscribed for the journal, for the material aid which, we think, they should unhesitatingly send us. If there are any who do not wish our journal, and do not intend to pay for it, such will please inform us, that we may be no longer at the expense of sending it to them. Those who, on the contrary, are pleased with our efforts, and who desire to see still greater improvements in our pages, will send us the small sum due us; and we promise that every cent shall be expended in making the Monthly the most valuable medical journal in the United States.

This number closes the Fifteenth Volume of the "Buffalo Medical Journal and N. Y. Review." With the June number, (which will be sent as an extra,) will be given the title-page and index; and with the succeeding, or July number, will commence the first number of the combined journal.

